# Phenotyping maize for adaptation to drought

**DOI:** 10.3389/fphys.2012.00305

**Published:** 2012-08-10

**Authors:** Jose L. Araus, María D. Serret, Gregory O. Edmeades

**Affiliations:** ^1^Facultat de Biologia, Unitat de Fisiologia Vegetal, Departament de Biologia Vegetal, Universitat de BarcelonaBarcelona, Catalonia, Spain; ^2^Independent ConsultantCambridge, New Zealand

**Keywords:** breeding, drought, maize, phenotyping, yield

## Abstract

The need of a better adaptation of crops to drought is an issue of increasing urgency. However, enhancing the tolerance of maize has, therefore, proved to be somewhat elusive in terms of plant breeding. In that context, proper phenotyping remains as one of the main factors limiting breeding advance. Topics covered by this review include the conceptual framework for identifying secondary traits associated with yield response to drought and how to measure these secondary traits in practice.

## General information

### Cultivated area and yield performance under optimal conditions

Maize is grown in virtually every country in the world, with a total production in 2002–2003 of 637,444,480 tons on 142,331,335 ha [Food and Agriculture Organization of the United Nations (FAO, 2007)]. This represents an average yield of 3.41 t ha^−1^, albeit very variable across countries. The United States of America and the People's Republic of China each produced over 100 million tons in 2002–2003, with US production being 2.25 times that of China. During the last decade, these two countries accounted for near 60% of total corn production. Six other countries produced at least 10 million tons during 2002–2003. These were, in order of production: Brazil, Mexico, Argentina, India, France and Indonesia. In 2020, demand for maize in developing countries is expected to exceed 500 million tons, and will surpass the demand for both rice and wheat (Pingali and Heisey, 2001). This projected rapid increase in demand is mainly explained by growth in the demand for maize as livestock feed (for poultry and pigs, particularly in East and Southeast Asia).

Genetic contributions to grain yield improvement in maize, attributable to plant breeding, have been estimated from studies which compare side-by-side the performance of hybrids and open-pollinated cultivars from various eras (Tollenaar and Lee, 2006). Most of the available literature concerns temperate maize and, with some reservations, may be applicable to tropical maize. Maize grain yield in the USA has increased by about 100 kg ha^−1^ year^−1^ or 2% year^−1^ from the start of large-scale adoption of hybrids by maize growers in the late 1930s until the first decade of the twenty-first century. About 75% of the yield improvement has been attributed to genetic gain and the rest to improved agronomical practices. The genetic gain was not associated with an increase in heterosis but rather with more stress tolerance (Duvick, 1999; Tollenaar et al., 2000) related to a higher leaf area per plant and higher harvest index (HI; Tollenaar and Lee, 2006). Two important physiological processes appear to be involved: (1) sustained leaf photosynthesis during grain-filling, which contributes to increases in dry matter accumulation; and (2) an increase in kernel number due to higher partitioning to the kernels during the sensitive period of kernel number determination. As a consequence, genetic gain is not associated with a change in HI because the increase in kernel number and the increase in dry matter accumulation during the grain filling period have been proportional.

The stability in HI rejects an increase in heterosis as being responsible for the genetic gain (Tollenaar and Lee, 2006). The higher dry matter accumulation in newer than in older hybrids during grain filling can be attributed, in part, to a longer duration of the grain-filling period in the former (Tollenaar and Lee, 2006). However, the silking date as well as the relative maturity do not differ between modern and old hybrids (Cavalieri and Smith, 1985), which further refutes changes in heterosis as responsible for genetic gain (Tollenaar et al., 2004). There is evidence that supports higher tolerance to low resource availability in newer maize hybrids; they performed better than older ones under stress, due to parental line involvement (Duvick, 1997) associated with better tolerance to high plant density (Tollenaar and Lee, 2006). In fact, plant water deficit will occur more readily at high rather than at low density, and resistance to high plant density involves resistance to drought stress when moisture becomes limiting (Tollenaar and Wu, 1999).

Anthesis-silking interval (ASI) under drought has become shorter in modern hybrids, and selection has possibly led to an increase in the growth of spikelets and ears and a reduction in final spikelet number (Bänziger et al., 2000). Moreover, “stay-green” or a reduction in the rate of leaf senescence during grain filling has been one of the traits that were the most visually distinctive between older and newer hybrids (Duvick et al., 2004a). Changes in constitutive traits such as plant phenology also seem to be involved in the different response to limiting resources. Older hybrids suffered a greater yield loss, in part, because they had extracted most of the plant-available water before entering the critical flowering period (Nissanka et al., 1997; Campos et al., 2004). In temperate maize hybrids there has also been a significant reduction in tassel size. From 1967 to 1991, tassel dry weight decreased by 36% (Duvick and Cassman, 1999). However, in tropical maize, the indirect pressure of selection to reduce tassel size by selecting for increased grain production has had relatively modest effects on tassel size. Therefore, tropical inbreds usually still possess a relatively large tassel, which may eventually have a negative effect on the development of ear and silk when the supply of photoassimilates is limited by drought stress (Ribaut et al., 2004; Sawkins et al., 2006).

Retrospective studies also show a large hybrid-by-environment interaction in terms of grain density. The genotype-by-environment interaction (GEI) could be a result of: (1) a greater genetic yield potential of newer hybrids; (2) a greater ability of newer hybrids to tolerate low resource availability; and (3) a greater general stress tolerance in newer hybrids (Tollenaar and Lee, 2006). Increased yield of newer hybrids could be a result of the synergistic effect between increased yield potential and increased resource availability (Duvick and Cassman, 1999). In general, increased yield potential will place a greater demand on all resources, resulting in increased stress frequency unless the greater yield potential is associated with an increase in general stress tolerance. In fact, yield stability and general stress tolerance are highly associated and yield stability does not appear to have declined with increasing yield potential (Tollenaar and Lee, 2002; Duvick et al., 2004b).

### Genetic and genomic resources

Hybrids tend to concentrate on a few inbred lines and their derivatives; less than 5% of the world's maize germplasm has been used by US breeders (Taba et al., 2004). In years to come, the ancestral base of US maize hybrids will increase as exotic germplasm is introgressed. Genetic diversity really is available to minimize the risk of a widespread catastrophe. Goodman (1998) has already shown a twofold increase in the use of exotic germplasm in a 12 year period from 1984 to 1996. In addition to having the right technologies, the other pillar of future breeding is to use more of the useful genetic variation that is available. This fact is of concern to all involved with maize germplasm, breeding and production (see Taba et al., 2004 for a comprehensive review). In that context, maize germplasm collections such as that hosted by CIMMYT (Centro Internacional de Mejoramiento de Maiz y Trigo; the International Maize and Wheat Improvement Center), which preserves genetic diversity and makes it fully available to all researchers with no restrictions on use, are the obvious source of genes for breeding efforts to develop tropical and subtropical maize better adapted to drought.

### Relevant results published in the area of drought adaptation

In general, average yields in tropical and subtropical regions are far lower than in temperate ones, with sub-Saharan Africa way below other regions with average values across countries of around 1 t ha^−1^. This is in spite the fact that maize is one of the main crops in these regions, where the effects of climate change including rising temperatures, evapotranspiration losses and, eventually, decreasing rainfall are expected to be particularly negative (World Bank, 2008). The possibilities for alleviation of water stress are limited. The majority of tropical maize is grown under rainfed conditions and poor farmers from these regions are unable to implement crop management strategies that might at least mitigate such constraints. In such a scenario, breeding for drought adapted maize remains the best alternative.

However, advances in breeding are frequently hindered by methodological bottlenecks. Among these, proper phenotyping is perhaps one of the most obvious today. This was not so evident few years ago, when phenotyping was considered as something already achieved, whereas emphasis was placed on other more fashionable breeding approaches such the adoption of molecular marker-assisted selection (MAS), genetic modification and the different “omics.” Fortunately, the situation seems to have changed and awareness is now increasing that new genetic and genomic tools will enhance but not substitute for the conventional breeding evaluation process (Varshney et al., 2005), and that only through an integrate use of different disciplines (including proper phenotyping) will breeding be speeded up. In that context, identification of key physiological processes associated with yield improvement and the determination of gene-to-phenotype associations can potentially increase the efficiency of breeding, whether through traditional or molecular methods (Araus et al., 2003, 2008; Tollenaar and Lee, 2006) including genomic selection propitiated by the availability of dense molecular markers (Crossa et al., 2010; Cabrera-Bosquet et al., 2012).

### Primary determinants of grain yield and drought adaptation

Grain yield may be expressed as the integrated response of different plant processes to a limiting resource such as radiation or water. Two main steps are involved: production of photoassimilates, and its further transformation onto an economic (usually harvestable) component. An additional factor to consider is the phenological stage of the plant when the limiting resource acts.

#### Radiation limited yield

Grain yield (GY) can be considered the product of the following:

GY=RAD · %RI · GLD · RUE · HI

where: RAD = incident radiation received per day (e.g., 20 MJ m^−3^); %RI = % intercepted radiation over crop life cycle (e.g., 50%); GLD = green leaf duration (e.g., 100 days); RUE = radiation-use efficiency, taken as 1.5 g MJ^−1^; HI = harvest index (0.45; range 0.4–0.55 under well-watered conditions). Thus:

$$\text{GY}=[\text{20}\cdot \text{0}.\text{5}\cdot \text{120}\cdot \text{1}.\text{5}]\cdot \text{0}.\text{45}={\text{810 gm}}^{\text{−2}},\text{or 8}.{\text{1 t ha}}^{\text{−1}}$$

Grain yield can be reduced by the effects of drought on most of these factors (Andrade et al., 1996). Drought during establishment can reduce plant germination, while water stress during leaf area expansion reduces leaf area and radiation interception. Later in growth, it will reduce green leaf duration from accelerated senescence, and reduce RUE by direct effects on photosynthesis (Dwyer et al., 1992). It can also have direct effects on yield components through induced barrenness, kernel abortion or shriveled grain, which can in turn reduce HI. The rate of seasonal dry matter accumulation is a function of interception and utilization of incident solar radiation. Differences in the rate of dry matter accumulation can be attributable to increased light interception due to: (1) greater maximum leaf area index (LAI); and (2) reduced leaf senescence (greater “stay-green”) during grain filling and a greater canopy-level efficiency of utilization of intercepted radiation, due to higher leaf angle and a reduced functional leaf senescence sustaining leaf photosynthesis during grain filling (Tollenaar and Lee, 2006). Reduction in leaf growth with water deficit may be coregulated with several mechanisms, each controlled by a large number of genes. Therefore, it may well be naïve to seek a single mechanism that accounts for the effect of water deficit on leaf growth and for the genetic variability of this process (Tardieu, 2006).

#### Water limited yield

Passioura (1977) proposed a parallel way of considering grain yield in a water limited situation:

GY=W · WUE · HI

where: W = water transpired by the crop (e.g., 400 mm); WUE = water-use efficiency, biomass/unit water transpired (e.g., 4.5 g m^−2^ mm^−1^). Thus:

$$\text{GY}=[\text{400}\cdot \text{4}.\text{5}]\cdot \text{0}.\text{45}={\text{810 gm}}^{\text{−2}},\text{ or 8}.{\text{1 t ha}}^{\text{−1}}$$

In the same sense, Blum (2006) summarized the primary factors responsible for superior performance of drought-adapted cereal cultivars, grouping them into four categories:

- capturing more soil water—thus, where deep soil moisture is available, deep-rooted cultivars demonstrate a clear yield advantage under drought (Lorens et al., 1987)
- economizing water use
- maintaining cellular hydration
- utilizing stem reserves for grain filling under stress—perhaps less applicable to maize than to small grain cereals.

#### Seedling establishment and pre-flowering growth

A requirement for high yield is an adequate plant stand. If drought severely reduces the stand at the onset of the season, farmers can replant fields with a shorter duration cultivar or a different species, although this entails additional cost. A limited research effort directed toward improving seedling establishment suggests that natural selection may have exploited most of the genetic variation for this trait. Recurrent selection based on stressed seedlings in the field showed only modest increases in survival under water deficit (Bänziger et al., 1997). Selection for improved survival and biomass production under post-emergence drought stress is also difficult because environmental variation is high in field screens. A recent study of the effects of pre-flowering growth on maize has demonstrated that this type of stress leads to significant reductions in plant height, in leaf area per plant and in grain yield, but to an increase in HI of several percentage points (Moser et al., 2006). However, the number of kernel rows was also reduced by stress prior to flowering, leading to a reduced kernel number per plant. Genotypes showing tolerance at flowering were not necessarily the most drought-tolerant in the pre-flowering phase. Early seedling vigor is a general expression of heterosis in cereals and is beneficial for reasons that may be related to reduced evaporation, thereby economising on water use.

#### Flowering

A failure of the rains later in the season when replanting is not possible may lead to a total crop loss, since maize yield in conventionally selected cultivars is often reduced two to three times more when water deficits coincide with flowering, compared with other growth stages (Shaw, 1977; Grant et al., 1989). Maize is thought to be more susceptible than other rainfed crops because of its near-synchronous development of florets, usually on a single ear, and because of the exposure of silks and pollen caused by the physical separation of male and female flowers on the same plant. Spikelets that are growing rapidly are more likely to set seed; one indicator of this is rapid silk extrusion. Since the date on which anthesis occurs is affected little by drought, slow silk growth results in a long ASI, a trait that is easily observed by breeders. A long ASI is an external indicator of a reduced partitioning to the ear, resulting in a slow spikelet growth rate (Edmeades et al., 2000b; Monneveux et al., 2006). Plants with a large ASI under drought are often barren, or have few grains per ear. Grain yield of maize grown under severe water stress at flowering is highly correlated with kernel number per plant (*r* = 0.90^**^) and quite strongly with ASI (*r* = −0.53^**^; Bolaños and Edmeades, 1996).

Factors affecting grain set under drought have been extensively reviewed by Westgate (2000). Grain number per plant in water-deficient maize appears to depend directly on the flux of current photosynthates during the 2 weeks bracketing flowering (Schussler and Westgate, 1995). It appears that reserves of pre-flowering assimilate are simply not attracted to the ear; perhaps the carbohydrate metabolism of the ovaries of water-stressed plants is disrupted, thereby impairing sink strength (Zinselmeier et al., 1995c; Westgate, 1997; Saini and Lalonde, 1998). However, once kernels enter the linear phase of biomass accumulation, they develop the sink strength needed to remobilize carbon reserves. This, along with continued photoassimilation, determines final kernel weight. The critical step in determining HI appears to take place 10–15 days either side of flowering. When assimilate flux per plant is reduced by competition, it has been shown that tassel growth is favored over ear growth (Edmeades et al., 2000a), and a similar tendency has been observed by Bolaños and Edmeades (1993a,b) under drought. Reductions in plant height and tassel size have also been associated with a reduction in ASI (Fischer et al., 1983, 1987). Although little is known about competing effects of root growth on ear growth, Bolaños et al. (1993) reported that, in one tropical maize population, reduced root biomass was associated with increased ear growth under drought.

#### Leaf growth and anthesis-silking interval

Leaf growth and ASI are the main determinants of source and sink strengths of maize, via their relations with light interception and HI, respectively. They depend on the ability of leaves and silks to expand under fluctuating environmental conditions, so the possibility is raised that they may have a partly common genetic determinism. This was tested in a mapping population segregating for ASI. For well-watered plants, the alleles conferring high leaf elongation rate conferred a low ASI (high silk elongation rate). Under water deficit, the allele for leaf growth maintenance was, in all cases, that for shorter ASI (maintained silk elongation rate). By contrast, other regions influencing ASI had no influence on leaf growth. These results may have important consequences for modeling the GEI and for designing drought-tolerant ideotypes (Welcker et al., 2007).

#### The relationship between anthesis-silking interval and grain yield

**Figure 1:**
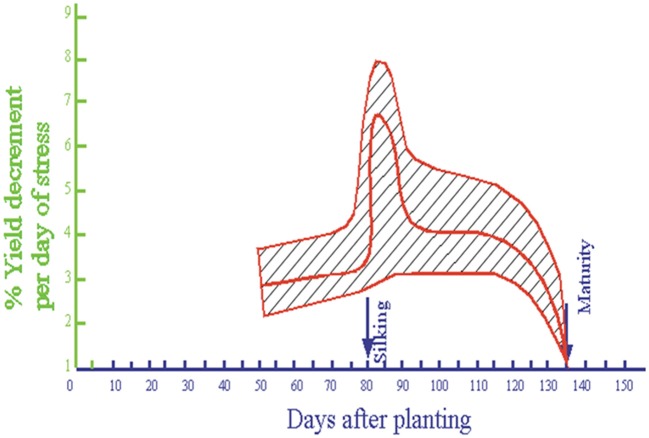
**Relationship between yield loss per day of stress and growth stage in a maize hybrid bred in the 1960s (Redrawn from Shaw, 1977)**.

Stress susceptibility varies considerably throughout the life cycle of the maize plant, and is greatest at flowering. Much of our conventional thinking on the degree of susceptibility to stress has been based on research published by Shaw (1977), in which stress-induced loss of yield per day was related to developmental stage in a hybrid that is now almost 40 years old (Figure 1).

There is good evidence that this marked susceptibility to drought stress at flowering has diminished with selection. However, there remains considerable genetic variation for tolerance to drought at flowering in modern commercial Corn Belt germplasm (Campos et al., 2004; Barker et al., 2005). One clear indicator of stress at flowering is a delay in silk exsertion in conjunction with very little or no delay in anthesis, giving rise to an easily observed ASI. Correlation analyses relating secondary traits to grain yield under drought stress at flowering in tropical germplasm show a close dependence of yield on kernel number per ear (KPE; up to *r* = 0.9), and moderate to strong associations of grain yield and KPE with ASI (*r* = −0.4 to −0.7; Bolaños and Edmeades, 1996). Others have reported similar correlations between ASI and grain yield in a wide array of germplasm (DuPlessis and Dijkhuis, 1967; Jensen, 1971; Bolaños and Edmeades, 1993b; Chapman and Edmeades, 1999; Monneveux et al., 2006). These are among the largest correlations of any secondary trait with grain yield under drought (e.g., correlation of grain yield under stress with stay-green: *r* = 0.3 to 0.5; with weight per kernel, *r* = 0.2 to 0.4; Bolaños and Edmeades, 1996), and emphasize the critical importance of the flowering process in establishing KPE and in stabilizing yield under stress.

Where stress is severe enough to induce barrenness, ASI is also highly correlated with the number of ears per plant (*r* = −0.5 to −0.7). Thus, ASI measured at flowering can predict a significant proportion of variation observed in grain yield that is only revealed 2 to 3 months later. These results are not confined to older hybrids or tropical germplasm. Evaluation of a representative sample of 54 modern precommercial Corn Belt hybrids has shown a correlation between grain yield and ASI across water stress levels of −0.72^**^, and between kernel number per plant and ASI of −0.71^**^ (Edmeades, 2002, unpublished data). Andrade et al. (2002) reported a common relationship between kernel number per plant and plant growth rate when both water and nitrogen supplies varied. Evidence of this nature led Edmeades et al. (2000b) to conclude that variation for stress tolerance at flowering exists, and that ASI is a convenient external indicator of this and may be a reasonable indicator of tolerance to reduced photosynthesis per plant at flowering arising from many causes.

The heritability of ASI is often slightly higher than that for grain yield, and several QTLs associated with this trait under drought stress have been identified (Ribaut et al., 1996). Other studies have subsequently identified similar regions and confirmed those originally identified in several other crosses (Welcker et al., 2007). These authors have also reported a QTL that colocalises for leaf elongation as well as for short ASI, suggesting a common genetic control or that turgor maintenance affects both. Marker-assisted backcrossing of some of these QTLs has demonstrated significant improvement in grain yield under flowering stress (Ribaut and Ragot, 2007). Managed drought stress environments, where stress is imposed by withdrawing irrigation during an otherwise dry growing season, are a highly effective means of exposing genetic variation for ASI in a repeatable, reliable manner (see Bänziger et al., 2000 for a useful practical guide to their use).

#### Selection for traits that govern kernel set

Given this strong relationship between ASI and grain yield and/or kernel number, can selection for these traits lead to greater yield stability when drought stress coincides with the flowering period? If so, what are the limits to progress, and are there concomitant penalties in non-stressed performance? The best current examples of selection are found in tropical germplasm. Here, selection for improved grain yield under drought stress at flowering, achieved mainly by emphasizing increased grain yield and reduced ASI and barrenness, resulted in gains per selection cycle in yield, ASI, ears per plant (EPP) and HI under severe stress. These gains averaged, respectively 100 kg ha^−1^, −1.1 day (or around 15°C day), 0.03 and 0.013 respectively (Edmeades et al., 2000a). There were also modest increases in the KPE. The increase in HI occurred under both stressed and unstressed conditions (Edmeades et al., 2000b). Similar results have recently been reported in another tropical population (Monneveux et al., 2006). Selection for more rapid silk emergence also improved tolerance to low nitrogen (Bänziger et al., 1999; Zaidi et al., 2004). Subsequently, Bänziger et al. (2005) reported that hybrids selected under managed stress using similar protocols significantly outyielded commercial hybrids in Southern and Eastern Africa by an average of 17% at yield levels in the 0–3 t ha^−1^ range, 11% in the 3–6 t ha^−1^ range and 4% in the 6–9 t ha^−1^ range.

**Figure 2:**
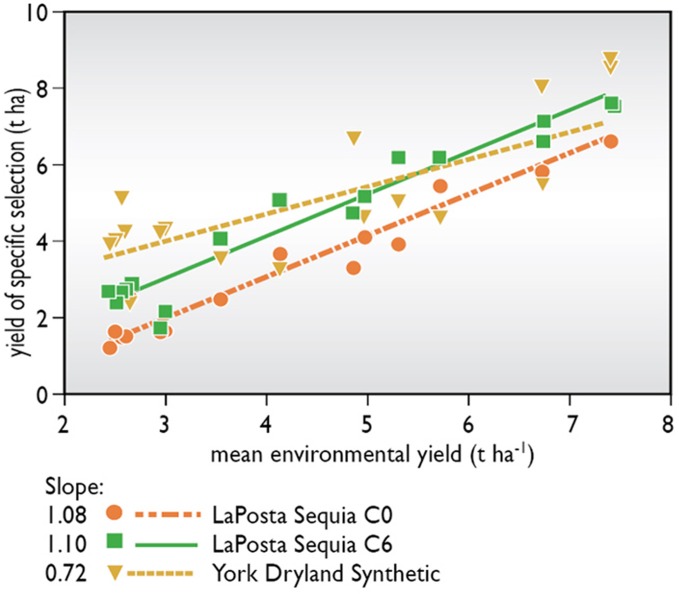
**Yield of unselected and selected versions of a tropical population, “La Posta Sequia,” when grown in environments to which it was not adapted.** Yields of an adapted Corn Belt population, “York Dryland Synthetic,” are given as reference.

Are the changes brought about by this type of selection subject to GEI? Tropical germplasm was largely selected in dry winter seasons where stress intensity and timing could be managed. Byrne et al. (1995) tested several selection cycles of tropical maize in the target environment, i.e., a normal summer crop season in a number of tropical sites away from the selection location. They found that 83% of the gains reported at the selection site carried over into the target environment. Pioneer Hi-Bred International Inc. tested initial and advanced selection cycles from three tropical populations, along with an older temperate drought tolerant population, at sites where the tropical germplasm was not adapted because of its photoperiod sensitivity. Although mean yields of tropical selections were not competitive with adapted temperate germplasm, gains due to selection for increased grain yields and due to reduced ASI and barrenness under stress were very similar to those observed at the selection site (Figure 2). These data suggest that changes due to selection targeted at the flowering period provide stability of performance across locations, even in locations where overall adaptation is poor. Zaidi et al. (2004) reported correlations between hybrids selected under drought versus conventional selection for yields under drought and under low nitrogen of 0.65^*^–0.67^*^ versus 0.44–0.46 ns, suggesting selection for tolerance at flowering reduced GEI. Reviews of progress in the ERA Corn Belt hybrid set, spanning improvement through pedigree breeding over the past 70 years, suggest that multilocation testing and screening at high plant densities have provided gains in yield in stressed and unstressed environments, although rates of gain in stressed environments were less than half of those in unstressed fields (Duvick, 2005; Campos et al., 2006).

#### Underlying causes for the relationship between anthesis-silking interval and grain yield

The dependence of kernels per ear on ASI suggests that germplasm that has not been previously exposed to strong selection under stress at silking may respond to stress by giving a large spread in silk emergence. Is an extended ASI a symptom of some deeper problem associated with spikelet fertility? There are three main reasons for the association between ASI, kernel set and grain yield (Hall et al., 1981; Otegui et al., 1995), as follows:

- *Lack of pollen because of heat, asynchrony, or because anthers do not exsert:* Hot dry weather during pollination may cause tassels to blast and kill the pollen before it is shed (Lonnquist and Jugenheimer, 1943; Schoper et al., 1987). Pollen quantity and viability are reduced in some genotypes when tassel temperatures reach 38°C (Lonnquist and Jugenheimer, 1943; Schoper et al., 1987), although drought *per se* does not appear to affect pollen viability (Hall et al., 1982; Schoper et al., 1986; Westgate and Boyer, 1986). Lizaso et al. (2003) have created algorithms that predict the effect of pollen viability on pollen concentrations considered critical for full kernel set, but have not provided any in situ measurements of pollen viability in the field. Asynchrony, caused by delayed silking, may simply result in a shortage of pollen for late emerging silks. Bassetti and Westgate (1994) have shown in one hybrid, P3790, that a reduction in kernel set occurred when pollen shed fell below 100 grains cm^−2^ d^−1^. This value agrees fairly well with that provided by Sadras et al. (1985), who reported that a mean pollen density of five grains per silk was necessary for 90% kernel set. Bassetti and Westgate (1994) also observed that this threshold pollen concentration increased if silks emerged more than 3 days after the start of anthesis. This suggested that the competence of silks and ovaries in late emerging silks, typically originating from the tip of the ear, had declined. In tropical genotypes that are usually characterized by large tassels, the period of shed is lengthened.
- Marked reductions in tassel size have occurred in temperate maize over the past 50 years of selection (Campos et al., 2006). However, in single cross hybrids that have been selected for high yield, tassels are typically half the biomass per plant of landraces. This means that the window of pollen availability is narrower, and the numbers of grains shed per day and per tassel are less. For example, Hall et al. (1982) cite pollen production per tassel of large open-pollinated varieties as 42.2 million versus 14.8 million for a Corn Belt synthetic. This can be compared with only 4.5 million pollen grains per tassel in modern hybrids in mid-Western environments (Westgate et al., 2003) and as little as 1.4 million per tassel in inbred lines (Fonseca et al., 2004), amounts that are undoubtedly affected by the environment (Uribelarrea et al., 2002). Male sterility can also be a cause of pollen shortage. Interplanting male sterile inbreds in varying proportions has been used as a means of altering pollen supply in quantitative studies of kernel set response to pollen supply (Lizaso et al., 2003; Westgate et al., 2003), and recurrent selection in populations for short ASI has sometimes resulted in a sharp increase in male sterile plants (Edmeades et al., 2000a).
- *Damage to the embryo sac during megasporagenesis:* This will normally prevent pollination, although silking may occur (Moss and Downey, 1971). Damage of this nature only occurs when severe water stress is encountered 1 to 2 weeks before silking, and is not reversible.
- *A slow rate of spikelet growth:* This results in a large ASI, silk senescence and abortion following pollination; drought reduces plant growth rate generally, and slows ear and spikelet growth.

Bolaños and Edmeades (1993b) found that selection for short ASI and increased grain weight under drought in a tropical population resulted in a significant increase in ear relative growth rate and a decrease in tassel relative growth rates. These changes are usually considered to reflect alterations in carbon partitioning. In this study, biomass of the upper ear at anthesis more than doubled over eight cycles of selection, and ear biomass per spikelet at anthesis increased by 12% per selection cycle. Rapid silk growth could be related to increased spikelet size, perhaps because there were fewer spikelets growing (Edmeades et al., 2000b; Monneveux et al., 2006). It is also possible that the earlier cessation of spikelet initiation in advanced selection cycles released already-initiated spikelets from a type of apical dominance, and permitted their more rapid growth.

The reduction in growth of tassels, stems, and roots that also accompanied selection probably released current assimilates to support accelerated ear growth. Reduced stem growth near flowering appears to accelerate ear growth, and results in reduced ASI (Sowell et al., 1961; Johnson et al., 1986; Edmeades et al., 2000b). Several recent studies have related kernel set to plant growth rate in the period of 10–15 days either side of flowering (Vega et al., 2001a), a technique that sharply reduces sampling errors. Lower plant and ear growth rates indicate lower assimilate flux to the growing plant and to the ear, a scenario that often results in kernel abortion within a few days after pollination (Schussler and Westgate, 1995). Increased rates of ear growth result in a rapid exsertion of silks, a higher rate of reproductive success, increased grain yield under all conditions, but especially under stress, and a general increase in HI (Bolaños and Edmeades, 1993a; Edmeades et al., 1999) in stressed and unstressed conditions. When slow growing silks of water-stressed plants were pollinated with fresh pollen, the majority of egg sacs were fertilized, but many ceased development 2 to 3 days after pollination (Westgate and Boyer, 1986; Bassetti and Westgate, 1993c). Others have also noted that when silks on plants exhibiting a long ASI are pollinated with fresh pollen, they will often not form grain (Lonnquist and Jugenheimer, 1943; Moss and Downey, 1971; Hall et al., 1981; Otegui et al., 1995). This failure probably reflects the state of health of the silks and the ovaries.

More recent research suggests that sucrose serves as a substrate for ovary growth, and that its concentration is a signal for gene expression (Boyer and McLaughlin, 2007). When the sucrose concentration is low, invertase genes are downregulated and genes associated with senescence are upregulated. Quantification of the extent of this type of abortion is difficult, since no trace of the aborted floret remains at maturity. It is possible that pollination with transgenic pollen, followed by testing for the presence of the transgene in specific kernel rings of immature ears with a quantitative polymerase chain reaction (PCR) assay, could detect abortion by comparing the position of the signal with that of filled kernels on mature unstressed ears. Infusion of sucrose into the internode near the point of ear insertion has been successful in reversing a large proportion of the grain loss associated with severe drought stress near flowering (Boyle et al., 1991). However, studies by Schussler and Westgate (1991b) and Zinselmeier et al. (1995a) both noted that there were direct effects of water stress on carbohydrate metabolism in the ovaries at silking.

In an elegant set of sucrose feeding studies, Zinselmeier et al. (1999) showed that ovary abortion under stress was related to the disappearance of starch reserves around the ovary walls. Both this and previous work showed that sucrose fed to stressed plants at flowering accumulated in the ovary tissues, and was apparently not broken down to hexose sugars in the first steps needed to form starch. It was hypothesized that water stress sharply inhibited the activity of acid invertase that catalyzes this step (Zinselmeier et al., 1995c, 1999).

**Figure 3:**
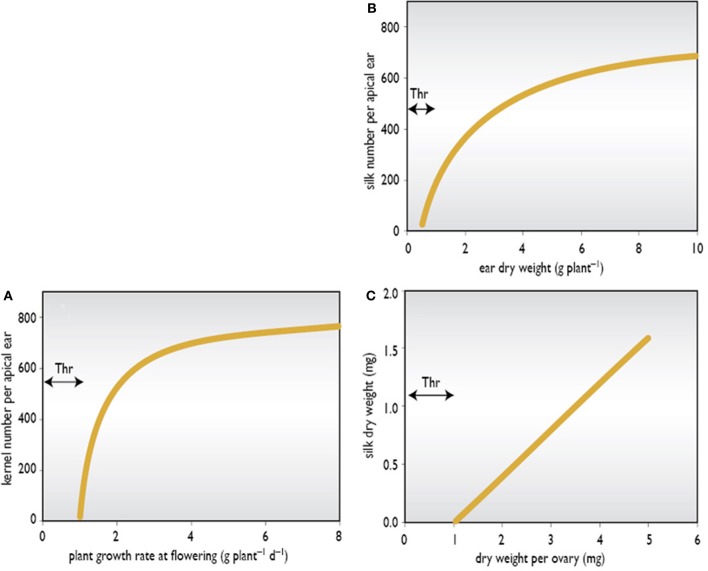
**Theoretical thresholds (Thr) in ear growth. (A)** Kernel number versus plant growth rate at flowering; **(B)** Silk number versus ear dry weight; and **(C)** Silk dry weight versus ovary dry weight.

It is apparent that the developing maize ear is a weak sink at a time when stem reserves of assimilate formed from previous photosynthesis are at a relatively low concentration (Westgate and Boyer, 1985). At silking, the ear appears unable to mobilize and attract these reserves and, instead, relies heavily on current photosynthesis (Schussler and Westgate, 1991a,b, 1994). This source of assimilate also supports concurrent stem, husk, tassel, and root growth (Zinselmeier et al., 1995b; Edmeades et al., 2000a). If this flux is reduced, or stems and tassels are growing aggressively, then the flux to the ear also falls, and kernel set can be reduced substantially. Therefore, accelerated silk emergence and a short ASI appear to be manifestations of increased partitioning of biomass to the developing ear and of a larger ear growth rate. Thresholds may be important. If assimilate flux to the ear falls below a certain threshold (Figure 3A), the normal pattern of silking is disturbed and the ear will abort completely or produce 30–50 kernels unevenly scattered over the rachis (Edmeades et al., 2000b).

Another threshold could be the ear dry weight needed to generate silk growth (Figure 3B). We also hypothesize that there is a threshold weight or growth rate for each ovary before silk growth will commence (Figure 3C). Non-destructive morphometric methods for estimating the thresholds of reproductive growth versus plant growth rate and kernel set versus ear growth rate have been described for maize by Vega et al. (2001a,b). They related kernel number per plant to plant growth rate around flowering to estimate threshold growth rates of the type illustrated in Figure 3A. Using similar methods in a series of hybrids released in Argentina over a 30 year period, Echarte et al. (2004) showed that the threshold plant growth rate for kernel set has fallen with selection, implying that modern hybrids can set kernels at lower plant growth rates than older hybrids.

Assuming that the methodology exists to estimate these thresholds precisely, it is very likely that genetic variation will be detected for all the thresholds described. Silks age, and if the silk has been emerged for 7 to 8 days, it will begin to senesce at its base. This will prevent the growth of pollen tubes to the ovary (Bassetti and Westgate, 1993a,b). When evaluating the effects of time of pollination on kernel set, Anderson et al. (2004) reported a rise in kernel set until 6–8 days after first silk, at a time when the maximum number of silks were exposed (Fonseca et al., 2004) and then a general decline in kernel set that, presumably, reflected senescing silks. The timing of the decline varied with year, suggesting that environmental conditions may affect the speed at which senescence occurs. When growth of silks is slowed by water stress early in their lives (e.g., 3 days after first silk), silk senescence is delayed. However, when the stress occurs a few days later, it serves to accelerate the senescence process (Bassetti and Westgate, 1993c).

#### Grain filling and stay-green

Provided that an ear has been established, the maintenance of a green functional canopy and a capacity to remobilize carbohydrates stored in the stem and husk should contribute to high yield under terminal drought stress. Associations between foliar stay-green and yield are often weak (Bolaños and Edmeades, 1996), and reasons for this must be sought in the nitrogen balance of the crop at that growth stage. Selection for more grains per plant will likely increase the internal demand for nitrogen and, since nitrogen uptake from a dry soil is low, this may result in “mining” of nitrogen from leaves, thus offsetting improvements in stay-green resulting from directed selection (Chapman and Edmeades, 1999). Duvick (2005) reported that stay-green had improved significantly over 50 years of breeding in Corn Belt maize, although the improvement was much greater under unstressed conditions than under terminal drought. However, QTLs have been identified in sorghum that significantly extend stay-green under drought (Harris et al., 2007), and it seems likely that they will also be identified under moderate terminal drought in maize.

Under drought stress, delayed senescence (commonly termed “stay-green”) during post-anthesis can sometimes be accompanied by maintenance of leaf water status, as in the case of stay-green sorghum (Xu et al., 2000). However in maize, stay-green was associated with higher yield (Ma and Dwyer, 1998), probably because of nitrogen use factors rather than plant water status effects (Blum, 2006). Thus, stay-green and kernel numbers are affected by nitrogen uptake and use efficiency, and by nitrogen remobilization (Gallais and Hirel, 2004). The most important single factor influencing nitrogen use efficiency is glutamine synthetase (Hirel et al., 2007). Other factors affecting stay-green are growth regulators. Thus, increasing the amount of endogenous cytokinin (Ori et al., 1999) or reducing the production of ethylene led to a delay in senescence (John et al., 1995). In fact, 1-methylcyclopropene has recently been commercialized for application in maize and other crops; it apparently binds with ethylene receptor sites in plants, reducing the negative effects of ethylene. In other cereals, ethylene has been related to decreased kernel number (Hays et al., 2007).

Irrespective of the underlying cause, stay-green may be a consequence of a plant's being able to keep a better water or nitrogen status rather than a primary factor in itself. Whatever the physiological mechanism involved in the adaptive trait, stay-green is a major factor that may contribute to improving grain yield when water shortage occurs during flowering and at the beginning of grain filling, provided that water is available further during grain filling (Ribaut et al., 2004). Regarding the use of stem reserves stored before and during heading for grain filling under stress, it seems that this characteristic is not evident in maize.

## Methodology

### Breeding strategy

#### Multilocation testing

Conventional breeding for drought tolerance based on extensive multilocation testing of progenies and GEI analysis has successfully increased grain yield under well-watered and moderately stressed environments. However, the use of nurseries where timing and intensity of water deficits are carefully managed, combined with the use of secondary traits, is more efficient and generally cheaper than multilocation testing (Monneveux and Ribaut, 2006). Proper control of the spatial variability inherent to field testing may also help to improve the efficiency of maize breeding for abiotic stresses (Cairns et al., 2012; Prasanna et al., 2012). Moreover to facilitate the full potential of molecular tools greater emphasis needs to be given to reducing the within-experimental site variability, application of stress and characterization of the environment and appropriate phenotyping tools (Masuka et al., 2012).

#### Empirical versus analytical breeding

Grain yield and its response to stress are highly complex traits involving a long-term (the full crop cycle) interaction between the environment and plant characteristics and regulatory pathways at different scales of organization (from molecular to the whole canopy). Empirical breeding, which is based on selecting directly by yield, has limited success under drought, due to large genotype-by-season and genotype-by-location interactions, which cause a low heritability of yield (Araus et al., 2002, 2008; Monneveux and Ribaut, 2006; Lopes et al., 2011; Prasanna et al., 2012). Alternatively, analytical breeding consists of the use of secondary traits to either complement phenotypic selection or eventually replace selection based on yield as the only phenotypic trait. This approach may improve the selection response because heritability of some secondary traits remains higher than that of yield, where those traits exhibit enough genetic variability, and are genetically correlated with yield.

Even if analytical breeding is not widely recognized, in practice, many breeders use secondary traits in addition to yield to improve the selection response. This kind of evolved empirical breeding has the concept of ideotype as a cornerstone and, de facto, integrates the concept of secondary traits. In a purely analytical breeding scheme, ideotype would be replaced by a selection index formulated based on the adjusted weight of the different traits considered during phenotyping (see below).

#### Farmer participatory approach

In addition to the above considerations, the optimal managed growing conditions (for the available water) of an experimental station are far different from the conditions prevailing in the fields of resource-poor farmers. In recent years, due to the lack of impact of traditional plant breeding approaches in low-income countries, there has been a movement toward the greater involvement of farmers in variety selection, the so-called “farmer participatory approach.” This may be considered as an adaptation of phenotyping protocols that have been discussed elsewhere (Bänziger et al., 2000; Sawkins et al., 2006).

### Trial planning

#### Choice and characterization of the testing environment

The difficulty in choosing appropriate selection environments has restricted breeding progress for drought tolerance in highly variable target environments. GEI are common under drought and make breeding progress difficult. GEI may originate from environmental variation in the timing and severity of water deficits, from genetic variation in flowering time, and from nutrient deficiencies and toxicities whose occurrence and severity interact with water deficits (Bänziger and Cooper, 2001). Also, high error variances such as induced by variable plant stand or variable soil water holding capacity are intrinsic to many field trials grown under drought, and impede selection decisions. Even though there is extensive evidence that selection under target stresses may accelerate breeding gains for stress environments (Bänziger et al., 1997), the difficulty of choosing appropriate selection environments, given a highly variable target environment, may limit the identification of superior genotypes (Cairns et al., 2012; Masuka et al., 2012; Prasanna et al., 2012). While breeding programmes in high-income countries may resort to real-time geographic information system (GIS) information for adequately weighting information from multienvironments trial (Podlich et al., 1999), those opportunities rarely exist in low-income countries because there is a lack of both real-time GIS information and resources for conducting a large number of multienvironment trials.

### Water stress management and characterisation

Phenotyping for drought performance is not just a matter of choosing the right combination of traits and measuring them at the right time. It is also necessary to cope with other sources of uncertainty relating to the need for suitable test sites with a drought cycle, irrigation system, and trained staff. Efficient phenotyping (frequently termed “precision phenotyping”) implies meeting two requirements. The first requirement is proper stress management of the agronomic conditions (including irrigation management and agroclimatic record) in order to impose as closely as possible the desired stress in terms of severity and occurrence during the crop cycle. For example CIMMYT has traditionally put emphasis on inducing drought stress around the time of flowering rather than at earlier stages of the crop cycle. The second requirement is to phenotype the critical traits using the right procedure and/or tools. The main principles of drought environment management have been described by Bänziger et al. (2000), and its successful translation to into practical breeding has been well illustrated in a recent study (Bänziger et al., 2005).

### Plant water strategy

When consider the response of any crop to drought stress, it is convenient to distinguish between moderate and severe stress. Yield under moderate stress conditions is highly dependent on the yield potential of the cultivar. For most cereals, moderate stress means a yield reduction of no more than about 50% compared with non-stress conditions, where drought resistance is less of an issue than is the yield potential of the cultivar (Araus et al., 2002, 2008; Blum, 2006). When yield is further reduced by stress to a level far below 50% of yield potential, then yield potential becomes irrelevant or even a liability, and a plant cannot yield well without some protection against this dysfunction. However, there is a range of growing environments where the combined effect of both factors eventually makes selection more complex (Sawkins et al., 2006). Therefore, germplasm screening in the absence of water stress as well as under stress environments is usually required. This approach, with the simultaneous use of selection under different contrasted environments, has been successfully implemented in sub-Saharan Africa, where hybrids developed by CIMMYT have outyielded hybrids from commercial companies (Bänziger et al., 2005).

Experience from drought-resistant cereal cultivation during a century of scientific breeding (Araus et al., 2002, 2004, 2008; Blum, 2005; Tollenaar and Lee, 2006) clearly indicates that drought resistance in crop plants at this level of stress is mainly derived from their ability to sustain tissue hydration under drought (i.e., dehydration avoidance), rather than an ability to sustain biological function when tissues are dehydrated (i.e., dehydration tolerance). However, until recently, most molecular biology approaches involving plant transformation, for example, have traditionally dealt with dehydration tolerance rather than avoidance (Araus et al., 2003). Dehydration avoidance in drought-resistant cereals cultivars is largely derived from constitutive traits (i.e., traits expressed in the absence of stress) rather than from drought-responsive traits (Blum, 2005). Constitutive traits may include seedling vigor, early, or synchronized flowering, leaf area, potential root length and plant size. In a historical perspective, the role of drought-responsive genes in comparison to genes that control constitutive traits seems to have had a relatively moderate role in the development of drought-resistant cereal cultivars, perhaps with the exception of osmotic adjustment (Blum, 2006), which does not seem to play an important role in maize (Tardieu, 2006).

### Phenotyping traits

For a secondary trait to be useful in a breeding programme, it has to comply with several requirements (Araus et al., 2002, 2008; Lafitte et al., 2003):

- It should be genetically correlated with grain yield in the environmental conditions of the target environment, i.e., the relationship with yield has to be causal not casual.
- It should be less affected by environment than grain yield is; i.e., it should have higher heritability than the yield itself, and so less GEI.
- Genetic variability for the trait must exist within the species.
- In the case of traits addressed in breeding for stress-prone environments, the trait should not be associated with poor yields in unstressed environments. Unfortunately the latter is the case for many traits selected because they confer tolerance instead of avoidance of a given stress (Araus et al., 2002, 2003).
- It should be possible to measure the trait rapidly, more economically than yield itself, and in a reliable way.
- The trait must be able to be assessed in individual plants or in very small plots, preferably by non-destructive means.

Most successful traits are “integrative,” either in time (reflecting physiological activities throughout the growing cycle), or in level of organization (i.e., at the whole plant level or, even better, at the level of the canopy), or both (Araus et al., 2002, 2008). In such a category we may include phenological traits (either constitutive or affected by stress) having an effect on HI (such as time to anthesis and ASI) or on energy uptake (stay-green), as well as other traits related with water status (such as transpiration and stomatal conductance).

#### Anthesis-silking interval

By determining genotypic correlations between a range of secondary traits and grain yield under drought, Bolaños and Edmeades (1996) found that reproductive traits related with HI, such as ASI, explained much more of the variation in yield than did traits related to plant water status, water use and WUE (e.g., leaf extension rate, canopy temperature, leaf erectness, leaf rolling, and leaf senescence). Indeed, ASI is one of the few examples of secondary traits widely used for maize selection under drought. The trait was developed by CIMMYT (Bolaños et al., 1993; Bolaños and Edmeades, 1996). ASI is an excellent secondary trait since it exhibits a significant negative correlation with grain yield and relatively high heritability, plus the other requirements indicated above. However, because continued selection for secondary traits results in changes in the underlying genetic correlation between traits, these relationships require reevaluation over time (Edmeades et al., 1997). Moreover, a short ASI has already been incorporated into the genetic background, which means that phenotyping for other traits is increasingly important. As noted above, there is a consistent correlation between ASI and kernels per ear under drought stress at flowering, normally ranging from −0.3 to −0.7. Gains have been made in yield and through reduced barrenness under stress when ASI has been used directly in selection.

Why not simply continue to use this trait as an integrated indicator of reproductive competence under stress? The following are some of the limitations of ASI as a selection trait:

- It does not capture variation in flowering behavior within and among plants. It is not clear what a plot value for ASI means at the individual plant level. Fifty percent anthesis and silking dates do not reflect the trajectory of anthesis or silking over time, but merely capture the median behavior of the population of plants. Thus, ASI does not describe attributes of a population of silks or pollen grains, nor can it quantify the asynchronous exsertion of silks within ears. It does not describe the fate of later emerging silks, nor the probability of these silks encountering pollen. ASI *per se* provides no information on changing spikelet numbers. Fewer spikelets appear to result in a greater reproductive efficiency per spikelet, but under unstressed conditions this reduction in spikelets may ultimately restrict yield potential, unless additional spikelets are added through a second ear per plant (Tollenaar et al., 1992).
- ASI is subject to error when silk delays are small. Since ASI is the difference of two measurements, both of which are subject to error, it can only be estimated precisely when the difference between anthesis and silking is reasonably large. Typically, errors in high-throughput visual estimation of flowering are ±1 day, so errors in ASI from rapid estimates are likely to be ±2 days. Other types of error can also occur when stress is severe. Some Corn Belt hybrids with small tassels enclose the tassel in the flag leaf during anther exsertion, and pollen shed cannot be observed. Similarly some hybrids will exsert silks in the gap between the stem and the ears leaf sheath, and are easily overlooked. ASI attains its greatest value for selection when it is >3 days, and when the exsertion of tassels and silks is clearly visible. Large ASI values of 5–8 days have no better heritabilities than shorter ASI of 3 days (Bolaños and Edmeades, 1996).
- ASI is time consuming to observe in the field. It is probably more economical to record the level of barrenness at harvest, provided the stress at flowering has been severe enough to induce barrenness in about 20% of the plants. The strong genetic correlation between ASI and ears per plant under severe drought stress (−0.7 to −0.9) can be used to advantage here (Bolaños and Edmeades, 1996).

We conclude that, while ASI continues to be a very useful trait that provides a snapshot of female versus male reproductive development, it does not provide useful information on the rate of silk appearance or the quantity of pollen shed per exposed silk.

#### Flowering parameters and kernel numbers

Details on applying these procedures are given in Bänziger et al. (2000). In brief, in the field they should be observed on a well-bordered area of known size in each plot, where there are no or very few missing plants.

- *Fifty percent silking and 50% anthesis and ASI*: Observe a known number of plants (guideline: *N* = 20 for inbred lines or hybrids; *N* = 35 for open-pollinated varieties) per plot at the same time each day until 50% of the plants in the plot have produced at least one visible anther or have at least one silk emerged, and record the date when this occurs. The ASI is: (days to 50% silk – days to 50% anthesis). Each of these parameters can also be expressed as heat units (°C day) if temperature data are being recorded near or in the plot.
- *Pollen density*: There are no known easy ways of measuring pollen production per genotype in plot sizes of less than 6 rows × 5 m that do not involve the slow process of bagging tassels to avoid cross contamination, followed by weighing the pollen shed into the bag each day. Pollen subsamples are then counted and weighed to provide an estimate of the number of pollen grains shed per plant (Hall et al., 1982). Aylor (2005) suggests that this method overestimates pollen at the silk level fourfold, because a proportion of grains lodge on leaves above the ear. The internal bag environment may also hasten anther dehiscence and increase shed. Where the goal is simply to measure the pollen density present in the plot from all shedding tassels (i.e., to assess if pollen is limiting seed set in a specific genotype growing in a trial) a sticky or liquid trap placed at silk level is usually used and is changed daily to avoid contamination with anthers, insects, etc. Counting is usually done either by suspending in an isotonic solution and counting with a Coulter counter (Fonseca et al., 2004), or by direct counting of the sticky surface using computerized imaging methods (Bassetti and Westgate, 1994; Fonseca et al., 2002; Uribelarrea et al., 2002).
- *Silk number*: Traditionally this has been counted by hand from approximately 10 ears per plot (hybrids; Bassetti and Westgate, 1993a). Usually, a cross section of the silk brush (1–2 cm long) is cut in the field and stored in water (for a few hours) or in 95% ethyl alcohol (several months). Where newly exposed silks need to be identified, the brush must be cut daily and the newly emerged silks visually identified by their bisected apical end (Cárcova et al., 2000). Similar methods were used by Uribelarrea et al. (2002), Cárcova and Otegui (2001), and Fonseca et al. (2004). Hand counting silk samples takes 10–15 min per sample and is, understandably subject to operator error. Computer imaging of pieces cut to a standard length seems increasingly feasible (Bassetti and Westgate, 1994).
- *Ears per plant*: The number of plants in a known area of plot are counted (*N* = 20 for inbreds or hybrids, *N* = 35 for open-pollinated varieties). At harvest, when ears are removed by hand, the number of ears with one or more kernels is counted. If there are no normal kernels on the ear, the plant is barren. When plots are mechanically harvested, ears are normally not visible, so counts must be made of ears that can be felt through the husk. Usually, this means that the ear needs to have ca 5 cm of grain formed along each of several ear rows, so that it can be felt through the husk as a solid mass. Ear numbers are recorded and divided by the number of plants for ears plant^−1^ and by the plot area for ears m^−2^.
- *Plant and ear growth rates at flowering*: The morphometric methods developed and described by Vega et al. (2001a) are recommended for this measurement, if thresholds of ear and plant growth for kernel set are required.

#### Measurement of source traits affecting individual kernel weight

These are largely related to the trait itself, or are measures of source (i.e., assimilate storage) activities:

- *Individual kernel weight*: When ears are being shelled, a representative sample of kernels is selected, either from the stationary sheller of from the grain stream of the plot combine harvester. Broken grains and non-grain matter are removed, and two aliquots of 100 representative kernels are each hand counted, dried to constant moisture at 80°C, and weighed. Alternatively, samples of about this number of clean representative kernels can be counted using an electronic seed counter, and weights of the samples taken as before. When using the average kernel weight obtained in this way to estimate kernels per ear, care must be taken to ensure that the moisture contents of all weights are compatible.
- *Stay-green*: This is usually assessed on a 0–9 scale, where each unit refers to 10% of the visually assessed foliage area that is green (or brown) at the time. This score is usually assessed on a plot basis once differences in foliar senescence of 2–3 units become clear among plots, and is usually repeated every 7–10 days until the leaves of about 10% of genotypes have fully senesced.
- *Remobilization of stem reserves*: Grain filling could continue in the absence of green leaf if assimilate stored in the stem and husk could be remobilized to the ear. Maize loses a significant amount of dry weight from both of these organs during grain filling, although taller maize plants with larger stem volumes are no more effective in maintaining kernel weight than their shorter counterparts when defoliated during grain filling (Edmeades and Lafitte, 1993). To measure remobilization *per se* requires an estimate of the loss of stem dry weight, either directly by destructive sampling, or from a reduction in stem diameter. Neither has been used on a large scale to assess differences among maize genotypes in remobilization capacity under drought; stable weight per kernel is the most economical way of estimating buffering capacity through remobilization.

The following measurements, carried out rapidly and precisely, are keys to successful genetic manipulation of kernel set and grain filling, and hence yield stability under drought stress:

- grain yield
- ASI
- number of silks emerging from stressed versus unstressed ears over time
- threshold plant growth rate for ear formation
- threshold ear size (or ear growth rate) for silk growth and for kernel set
- adequacy of pollen supply (its timing, intensity, and viability)
- ears per plant (or, conversely, barrenness)
- kernels per ear
- weight per kernel
- degree of kernel abortion in the first 5 days after pollination
- canopy stay-green estimates.

#### Water status parameters

**Figure 4:**
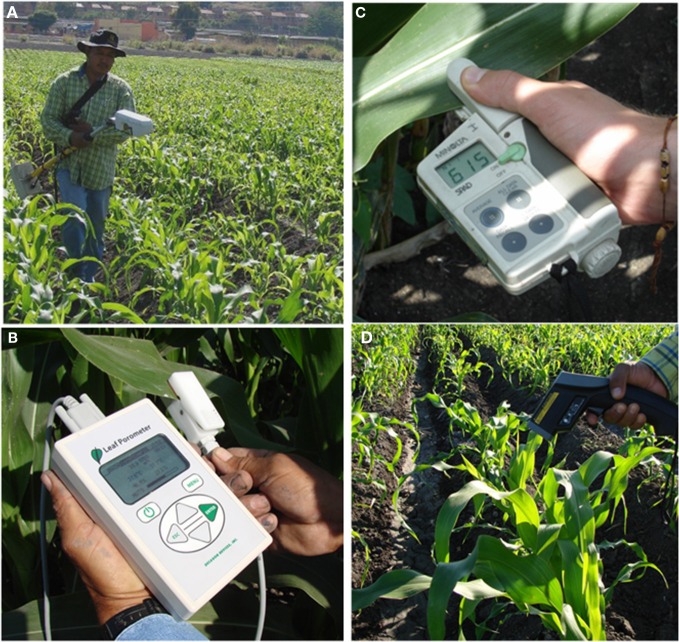
**Different devices to evaluate plant growth, phenology and water status. (A)** spectroradiometer with active sensor to measure the normalized difference vegetation index (NDVI); **(B)** porometer to measure stomatal conductance; **(C)** leaf chlorophyll meter; **(D)** infrared thermometer to measure leaf temperature.

The water status of the crop may be assessed through transpiration, i.e., the water used by the leaf or the plant. The rationale is quite straightforward: the better the status the more the plant will transpire. There are different potentials tools (or surrogates) that allow transpiration to be measured indirectly:

- *Porometry*: transpiration may be broken down into two components. One is the leaf conductance (mostly determined by how open the stomata are, i.e., the stomatal conductance, *g*_s_) which really depends on the water status of the plant. The other is the evapotranspirative demand, which depends on environmental variables such as temperature, relative humidity and wind. Thus, *g*_s_ may be used to screen for water status in maize (Sanguineti et al., 1999), and the current generation of relatively low-cost (a few thousand US$) and easy-to-handle porometers such as the Decagon Leaf Porometer SC-1 or the Delta-T AP4 allow rapid (20–30 s) measurement of leaf conductance (Figure 4). However, unless several porometers are used simultaneously, it may still be impractical for a large scale evaluation.
- *Canopy temperature*: Depression of the canopy temperature reflects evaporative cooling of the leaf surface due to transpiration. Measurements are performed from a distance using infrared thermometers (Figure 4), which are inexpensive devices (a few 100 US$). They are frequently used on crops with homogenous canopies (e.g., cotton or small grain cereals such as wheat or barley) provided that they fully cover the soil (Reynolds et al., 2001), the atmospheric conditions are adequate (sunny days, lack of wind, high evapotranspirative demand), and there are not strong differences in phenology (e.g., heading time for cereals) between genotypes. The canopy temperature has also been measured in maize (Sadler et al., 2000; Wanjura and Upchurch, 2000). However, the characteristics of the plant make it less practical to measure temperature at the canopy level, although it is possible to do it for individual leaves (Sanguineti et al., 1999; O'Neill et al., 2006), provided that they are fully exposed to the sun and at a similar angle.

**Figure 5:**
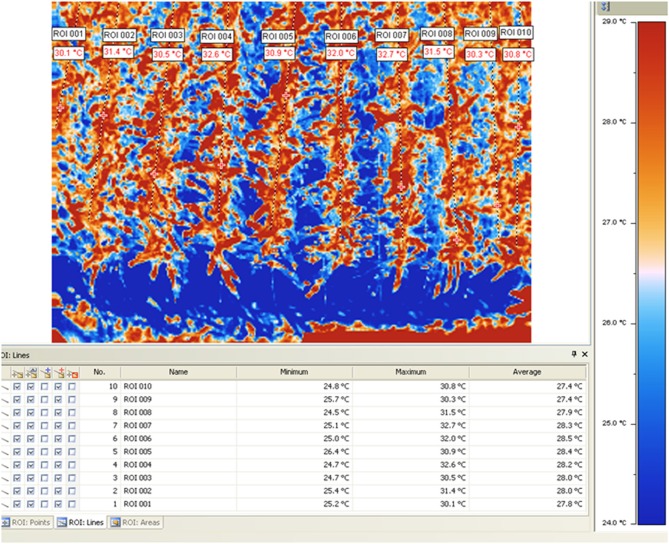
**Thermal image and corresponding temperatures of different maize testcrosses measured at CIMMYT's experimental station of Tlaltizapan (Ed. Morelos, Mexico).** For more information about the procedure used see Romano et al., 2011 and Zia et al., 2012.

New remote-sensing tools based on the use of thermal imaging to estimate plant water status at field level are achieving increased importance (Chaerle et al., 2007; Grant et al., 2007; Möller et al., 2007). Recently the use of thermography (Figure 5) has been proposed for high throughput phenotyping of tropical maize adaptation in water stress (Romano et al., 2011; Zia et al., 2012).

#### Oxygen isotope composition

The stable ^13^C/^12^C isotope composition (δ^13^C) measured in plant matter has been used to help breeding for drought adaptation in wheat and other small grain cereals. However, in maize, its C_4_ metabolism prevents the use of δ^13^C as a tool for screening (Monneveux et al., 2007; Cabrera-Bosquet et al., 2009b). The stable ^18^O/^16^O isotope composition of plant organic material (δ^18^O) has been shown to reflect the isotope composition of soil water, evaporative ^18^O enrichment in transpiring leaves, and isotopic exchange between oxygen atoms in organic molecules and local water in the cells in which the organic molecules are formed (Barbour, 2007). As plant material has been shown to record leaf evaporative conditions, measurement of ^18^O enrichment of the plant matter compared with the source water may provide a powerful tool for plant breeders (Barbour, 2007; Cabrera-Bosquet et al., 2009a). Although an integrative record of *g*_s_ may, in its own right, be of interest to breeders, the link between δ^18^O and crop yield is likely to stimulate greater interest.

**Figure 6:**
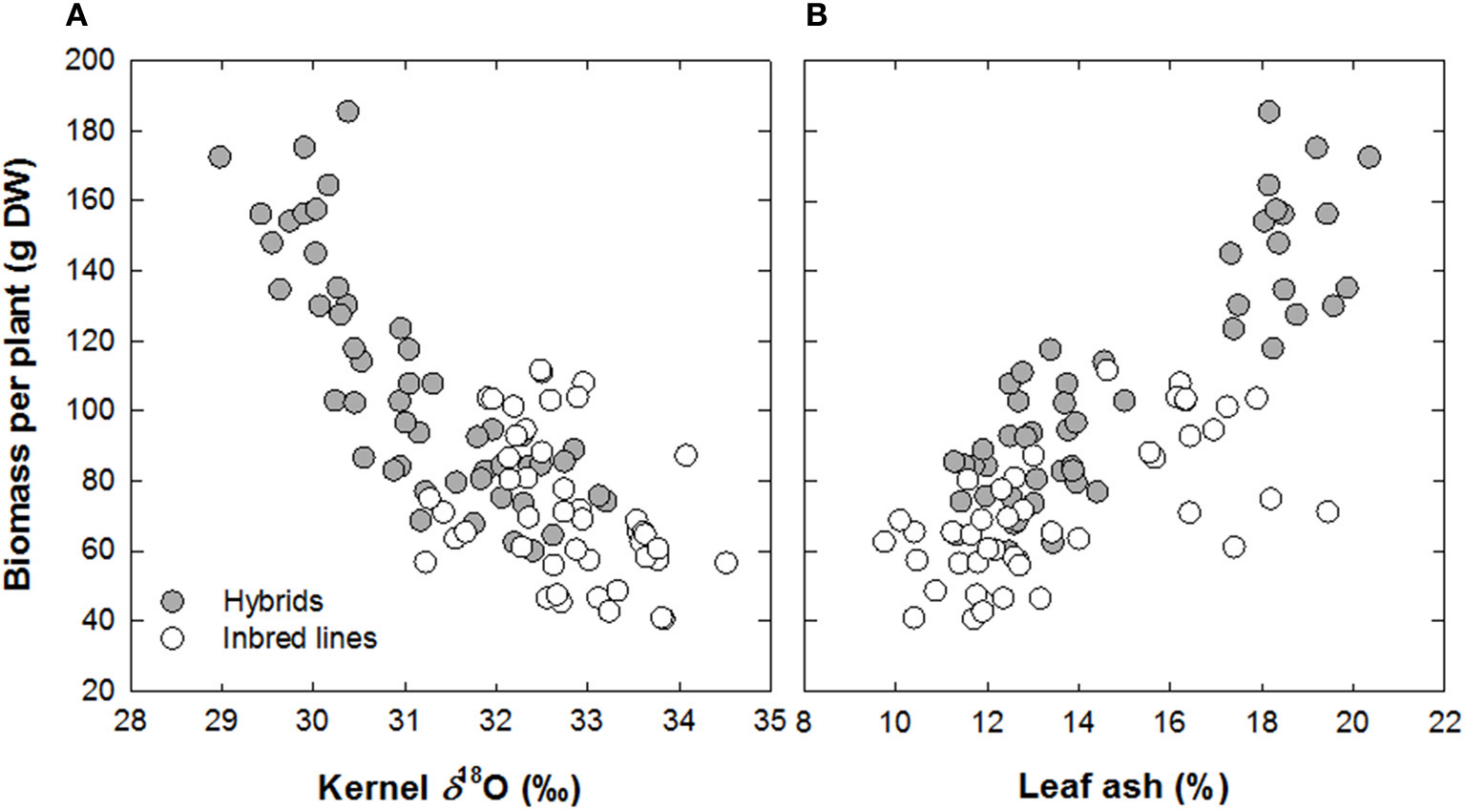
**Relationship between biomass per plant about 2 weeks after anthesis with **(A)** oxygen isotope composition in mature kernels (δ^18^O) and **(B)** ash concentration in leaves about 2 weeks after anthesis. Data from a set of maize inbred lines and derived hybrids grown under three different water regimes were plotted together (*n* = 96). Each point represents a mean value for three plots of a single genotype grown under a particular water regime (Redrawn from Araus et al., 2010)**.

Cotton and wheat display strong correlations between *g*_s_ and yield when grown in non-limiting environments (Lu et al., 1994; Sayre et al., 1997). Barbour et al. (2000) have shown that the δ^18^O of both whole leaf tissue and cellulose is strongly negatively related to the seasonal mean *g*_s_ and to grain yield for field-grown wheat. Therefore, the δ^18^O composition of plant tissue is of interest to breeding for improved water use and yield in crop species. Its theoretical foundations already seem reasonably well established (Farquhar et al., 2007), which may help its further adoption as a breeding tool. Some contradictory results (Sheshshayee et al., 2005) still need to be resolved, however, and practical aspects rather than theoretical ones prevent a more widespread adoption of δ^18^O as a breeding tool. First is its cost, which is still far higher than for δ^13^C, and second is the fact that, except for kernels, it is better to analyze chemical fractions such as cellulose rather than dry matter as a whole. In such a context, other surrogates for transpiration may be used such as the total mineral content accumulated in transpiring organs. For crops such as wheat and barley, these have shown a good positive relationship with grain yield (Araus et al., 1998; Voltas et al., 1998). Recently the potential utility of δ^18^O analyzed in kernels (Cabrera-Bosquet et al., 2009c) as well as total mineral (i.e., ash) content in mature but not senescent leaves (Cabrera-Bosquet et al., 2009b) has been demonstrated in maize. These approaches have also shown that phenotypic expression of heterosis in maize in linked to a better water status of hybrids compared with lines regardless of the growing conditions (Araus et al., 2010; Figure 6). While a potential limitation of a wider use of δ^18^O arise in its cost and technical facilities required, a recent study concluded that near infrared reflectance spectroscopy (NIRS) can be used as a rapid, cost-effective, non-destructive method for screening δ^18^O, moreover to represent an accurate method for predicting ash and N contents in the same samples (Cabrera-Bosquet et al., 2011). Therefore, these NIRS-based analytical methodologies represent a promising application in crop management and maize breeding programs for improved water and nitrogen use efficiency and grain quality.

#### Plant growth, senescence, and other traits: spectroradiometrical techniques

Extensive phenotyping of large field trials for several traits is extremely expensive. Spectroradiometrical techniques allow fast and non-destructive evaluation of different characteristics of plants. They, therefore, present opportunities to develop novel phenotyping platforms that allow large screenings of genotypes for several traits in multilocation field trials (Aparicio et al., 2000; Araus et al., 2001; Babar et al., 2006). These techniques allow monitoring of several dynamic complex traits with high temporal resolution (Araus et al., 2001).

The most common use of spectroradiometrical techniques is for evaluation of chlorophyll content and related traits (such as nitrogen content, green area), based in a shift of light absorbed in the visible (400–700 nm wavelength, where the photosynthetic pigments absorb) versus the near infrared bands (700–1000 nm) of the spectrum. The same principle is used to evaluate plant status at different organization levels (Figure 4), from the leaf (e.g., the portable leaf chlorophyll meter like SPAD, which works using the light transmitted) to the canopy (with land-based portable spectroradiometers), where the light reflected is usually measured and vegetation indices subsequently calculated, or even to the entire crop or ecosystem (with aerial or satellite placed sensors). One of the most common vegetation indices is the normalized difference vegetation index (NDVI), which may be used to evaluate crop characteristics such as early vigor and stay-green that may be important in maize, and even grain yield (Lu et al., 2011). The use of NDVI has also been proposed as a covariate trait to remove the effect of confounding management problems (e.g., differences in plant emergence across plots) on genotype grain yield performance (Bänziger, personal communication). Besides vegetation indices, other spectral indices allow the evaluation of different traits related to photosynthetic efficiency and water status. A list of the main spectral reflectance indices potentially useful in breeding programmes is summarized by Araus et al. (2001). In addition, recent development of new formulations of the water index (WI) may open up promising perspectives for its use in drought phenotyping (Babar et al., 2006).

Canopy spectral reflectance sensors have been grouped into two categories, active and passive. Active sensors (equipped with their own source of radiation) are less influenced by environmental conditions but measure few wavelengths (Teal et al., 2006; Marti et al., 2007). The most widely known example of a land-based portable spectroradiometer with these characteristics is the “GreenSeeker”^1^. Passive sensors (using solar radiation) are largely influenced by environmental conditions, but measure a wide spectral range with high spectral resolution (with a bandwidth of ca 2 nm; Araus et al., 2001; Osborne et al., 2002; Babar et al., 2006). The cost of active sensors is far less than (about quarter of) that of commercialized passive sensors and they are more suitable for phenotyping multilocation field trials because data collection can be performed during a more extended time than with passive sensors. Moreover, they are configured to faster (more automatic) collection of data. However, the few measured wavelengths and low spectral resolution of active sensors might limit the prediction of complex traits; they usually measure NDVI. Therefore, the development of active sensors with increased spectral range and resolution will certainly bring forward the application of canopy spectral reflectance as a component of high-throughput phenotyping platforms.

At the leaf level, in addition to the portable chlorophyll meters developed about 20 years ago and widely used currently to evaluate differences in leaf senescence or nitrogen status, there is a new generation of sensors specifically designed to evaluate other pigments like anthocyanins (e.g., Dualex 3.3 ANTH) and flavonoids (e.g., Dualex 3.3 FLAV)^2^. Their cost, even though about one third of that of multispectral passive sensors, still prevents their wide adoption in breeding programmes.

Other more novel phenotyping techniques based on chlorophyll fluorescence (Chaerle et al., 2007), digital imaging (Casadesús et al., 2007), or even the use of spectroradiometers covering the region of 2–3 μm wavelength are promising, although still very expensive and at an early phase of their development.

Many new phenotyping tools based on remote sensing are now available including non-destructive measurements of growth-related parameters and even grain yield predictions based on spectral reflectance (Weber et al., 2012). The ability to accurately estimate grain yield using spectral reflectance measurements prior harvest could be used to reduce phenotyping time and costs. Thus in a recent study with tropical and subtropical maize grain yield of 300 maize testcrosses grown under different water and temperature regimes was predicted using spectral reflectance (495–1853 nm) of both leaves and canopy measured between tassel emergence until milkgrain stage and using partial least square regression (PLSR) was used for data analysis (Weber et al., 2012).

#### Is it worth measuring metabolic levels?

The role of abscisic acid (ABA) in relation to drought has been intensively studied in maize over many years (Settler, 2006). ABA is widely believed to be a major contributor to the control of plant transpiration and leaf growth (Tardieu, 2006). Moreover, ABA is thought to inhibit cell division in the endosperm; if this occurs at an early stage, the kernels will abort. A lot of research has been undertaken on the control of biosynthesis and catabolism of ABA, and the action and role of ABA under water stress (Sawkins et al., 2006; Settler, 2006). The signaling pathways of ABA and ethylene overlap because mutants affected in their sensitivity to ABA are allelic with mutants of ethylene sensitivity (Beaudoin et al., 2000). Furthermore a similar overlap is observed between the signaling pathways of ABA and of sucrose (Leon and Sheen, 2003). However, this avenue, like others dealing with transient levels of metabolites and other growth regulators, has an inherent potential limitation. In the case of ABA, it provides just a measure of drought stress at the time of sampling and in the organ sampled. Moreover, the adaptive (i.e., positive) role of ABA is under challenge. In maize, near isogenic lines (NILs) have been produced for *root-ABA1*, a major QTL that affects root architecture, ABA concentration, and grain yield across different water regimes (Giuliani et al., 2005; Landi et al., 2005). The lines producing more ABA were those showing less yield performance not only under well irrigated conditions, but also under moderate water stress.

Carbohydrates are also claimed to be another critical control factor. The supply of photoassimilates to the developing maize grain is of critical importance during conditions of water stress (Settler, 2006; Tiessen et al., 2006). Carbohydrates, along with other compatible solutes may play a role in osmotic adjustment (OA), maintaining turgor pressure in cells (in leaves as well as in reproductive organs) during water stress. Tang and Boyer (2002) observed a decrease in osmotic potential of about 1.5 MPa in growing maize tissues subjected to water deficit, whereas Bolaños et al. (1993) observed a small OA in the same species. However, several studies in maize found no correlation between accumulation of osmolytes and yield (Bolaños and Edmeades, 1991; Guei and Wassom, 1993). In fact, OA may be incomplete in maize leaves subjected to mild air or soil water deficits (Bouchabke et al., 2006). Nevertheless, osmolytes may still have a role in plant survival, helping to maintain the reversibility of cell dehydration. In fact, osmolytes can also serve as antioxidants and chaperons.

A recent study in tropical maize has shown that different organs possessed distinct metabolite compositions, with the leaf blade displaying the most considerable metabolome changes following water deficiency. However whilst a general increase in metabolite levels under drought stress was shown, including changes in amino acids, sugars, sugar alcohols, and intermediates of the TCA cycle, these changes were not differential between maize hybrids that had previously been designated based on field performance as either drought-tolerant or susceptible. Nevertheless several metabolites displayed conserved responses to drought (Witt et al., 2011).

#### How to use phenotypic traits

Once diverse phenotypic data have been collected, the question arises as to how to use them. Valuable traits may be combined in a selection index which is, in a way, a quantitative translation of the ideotype concept. Fischer et al. (1989) have already obtained higher yield gains under severe moisture stress conditions in maize by using a selection index combining ASI, relative leaf extension and leaf death score, rather than selecting by yield *per se*. More recently, Bänziger et al. (2000) have proposed to combine data on stressed and unstressed yield, ASI, barrenness, and stay-green under stress in a selection index used by CIMMYT to identify superior genotypes with increases in yield averaging 100 kg ha^−1^ per selection cycle. When defining a selection index, weights of different traits are chosen based on variance and heritability and on genetic correlation with yield. In maize, weights typically allocated to secondary traits are +3, −2, −2, −2, and −1 for ears per plant, ASI, leaf senescence, tassel size, and leaf rolling, respectively (Bänziger et al., 2000). Selection indices are continuously redefined, giving attention not only to the target environment for selection, but also with a view to incorporating new secondary traits and innovative tools for their evaluation. Selection indices still have an important empirical bias related to the assigned weights of each of the phenotypic traits considered. A step forward would consist of integrating phenotypic data into a crop model. Models may help to manage phenotyping more efficiently. However, available models are not yet developed well enough to predict differences in performance across genotypes in a reliable manner. Nevertheless more recently a selection index method based on Eigenanalysis and developed by CIMMYT (Cerón-Rojas et al., 2006) has been proposed to calculate the best selection indices for each target environment.

## Conclusions

When drought occurs around flowering, grain number and, consequently, grain yield are affected markedly, particularly in maize. By contrast, losses due to drought during plant establishment are relatively low and can, to some extent, be offset by replanting. Therefore, research on traits affecting inflorescence and grain formation is and will continue to be a main priority in the tropical maize breeding research agenda at CIMMYT (Edmeades et al., 2000b; Bänziger et al., 2006). In such a context, productivity-enhancing traits become more important during flowering and grain filling. If terminal drought is the major constraint, then traits affecting grain filling (e.g., current photosynthesis, stay-green) will be more important (Monneveux and Ribaut, 2006; Monneveux et al., 2008).

### Conflict of interest statement

The authors declare that the research was conducted in the absence of any commercial or financial relationships that could be construed as a potential conflict of interest.

## Footnotes

^1^http://www.ntechindustries.com/greenseeker-home.html

^2^ftp://ftp.dynamax.com/DynamaxPDF/Dualex.pdf
